# The relationship between early life modifiable risk factors for childhood obesity, ethnicity and body mass index at age 3 years: findings from the Born in Bradford birth cohort study

**DOI:** 10.1186/s40608-015-0037-5

**Published:** 2015-02-24

**Authors:** Lesley Fairley, Gillian Santorelli, Debbie A Lawlor, Maria Bryant, Raj Bhopal, Emily S Petherick, Pinki Sahota, Darren C Greenwood, Andrew J Hill, Noel Cameron, Helen Ball, Sally Barber, John Wright

**Affiliations:** Bradford Institute for Health Research, Bradford Teaching Hospitals NHS Foundation Trust, Duckworth Lane, BD9 6RJ Bradford, UK; Faculty of Health Studies, University of Bradford, Bradford, UK; Clinical Trials Research Unit, University of Leeds, Leeds, UK; MRC Integrated Epidemiology Unit at the University of Bristol, Bristol, UK; School of Social and Community Medicine, University of Bristol, Bristol, UK; Centre for Population Health Sciences, University of Edinburgh, Edinburgh, UK; Institute of Health and Well-being, Leeds Metropolitan University, Leeds, UK; Division of Biostatistics, University of Leeds, Leeds, UK; Institute of Health Sciences, Leeds University School of Medicine, Leeds, UK; School of Sport, Exercise and Health Sciences, Loughborough University, Leicestershire, UK; Parent-Infant Sleep Lab, Department of Anthropology, Durham University, Durham, UK

**Keywords:** Body mass index, Overweight, Early childhood risk factors, Ethnicity, Born in Bradford

## Abstract

**Background:**

Many modifiable risk factors in early infancy have been shown to be associated with childhood overweight and obesity. These risk factors have not been studied within children of South Asian origin in the UK. The aims of this paper are to describe differences in the prevalence of modifiable risk factors for childhood obesity between children of White British and Pakistani origin and investigate the association between these risk factors and childhood BMI measured at age 3 years. We used data from a sub-study of the Born in Bradford birth cohort with detailed follow-up visits throughout early childhood. 987 participants with a BMI measurement at age 3 were included; 39% were White British, 48% were of Pakistani origin and 13% were of other ethnicities. Linear and Poisson regression models were used to assess the association between risk factors and two outcomes at age 3; BMI z-scores and child overweight.

**Results:**

Compared to Pakistani mothers, White British mothers were more likely to smoke during pregnancy, have higher BMI, breastfeed for a shorter duration and wean earlier, while Pakistani mothers had higher rates of gestational diabetes and were less active. There was no strong evidence that the relationship between risk factors and BMI z-score differed by ethnicity. There were associations between BMI z-score and maternal smoking (mean difference in BMI z-score 0.33 (95% CI 0.13, 0.53)), maternal obesity (0.37 (0.19, 0.55)), indulgent feeding style (0.15 (−0.06, 0.36)), lower parental warmth scores (0.21 (0.05, 0.36)) and higher parental hostility scores (0.17 (0.01, 0.33)). Consistent associations between these risk factors and child overweight were found. Mean BMI and the relative risk of being overweight were lower in children of mothers with lower parental self-efficacy scores and who watched more hours of TV. Other risk factors (gestational diabetes, child diet, child sleep, child TV viewing and maternal physical activity) were not associated with BMI.

**Conclusions:**

Whilst the prevalence of risk factors that have been associated with childhood greater BMI differ between White British and Pakistani the magnitude of their associations with BMI are similar in the two groups.

**Electronic supplementary material:**

The online version of this article (doi:10.1186/s40608-015-0037-5) contains supplementary material, which is available to authorized users.

## Background

Risk of obesity begins in early childhood; in England over a fifth of children are overweight or obese at school entry (aged 4–5 years) [1]. At this age the prevalence of obesity is higher in children of South Asian origin at 10.4% compared to the national average of 9.5% [1].

At birth and in early infancy there is an inverse association between body mass index (BMI) and adult coronary heart disease risk, however from age seven years a positive association emerges that strengthens to adolescence and early adulthood when the magnitude is similar to that seen for BMI measured in mid-life [2,3]. These studies have however, largely been conducted in White European origin populations. South Asian adults are at increased risk of diabetes and cardiovascular disease [4-6] and South Asian children have been shown to have a greater fat mass for a given BMI than their White European counterparts [7]. Furthermore markers of diabetes and cardiovascular disease risk are increased in South Asian children and adolescents [8,9].

It is important to understand whether modifiable characteristics are related to BMI in early life in order to ensure that appropriate interventions are developed to promote maintenance of healthy BMI levels into mid-childhood, when important relationships with future coronary heart disease risk emerge. Furthermore, knowing whether associations differ between South Asian and White European origin infants is important in knowing whether interventions should target different risk factors in these groups in order to reduce the ethnic differences in risk. Epidemiological evidence has highlighted the importance of a number of exposures in pregnancy and early life that are associated with the development of obesity in childhood. Risk factors that have been associated with increased risk of childhood overweight and obesity include maternal smoking [10-12], maternal diabetes [10], maternal pre-pregnancy overweight [11,13], infant feeding (including breastfeeding duration, age at introduction to solids and dietary intake) [10,11,14], parenting styles [15,16], sleep duration [10], sedentary behaviour (including TV viewing time) and physical activity [10,13]. The prevalence of these modifiable risk factors and their association with obesity have not been studied in pregnancy and early childhood within children of South Asian origin in the UK.

There are two aims of this paper; (i) to describe differences in the prevalence of potentially modifiable risk factors for childhood obesity between children of White British and Pakistani origin and (ii) to investigate the associations between these risk factors and childhood BMI at 3 years of age.

## Methods

### Sample and participants

Born in Bradford (BiB) is a longitudinal multi-ethnic birth cohort study aiming to examine the impact of environmental, social, psychological and genetic factors on maternal and child health and wellbeing [17]. Bradford is a city in the North of England with high levels of socio-economic deprivation and ethnic diversity. Approximately half of the births in the city are to mothers of South Asian origin. The majority of women were recruited while waiting for their glucose tolerance test, a routine procedure offered to all pregnant women registered at the Bradford Royal Infirmary, at 26–28 weeks gestation. For those consenting, a baseline questionnaire was completed via an interview with a trained study administrator. The full BiB cohort recruited 12,453 women during 13,776 pregnancies between 2007 and 2010 and the cohort is broadly characteristic of the city’s maternal population. The participants gave informed consent for the data collection and ethical approval for the data collection was granted by Bradford Research Ethics Committee (Ref 07/H1302/112).

A subsample of the BiB cohort (BiB1000) recruited between August 2008 and March 2009 were invited to participate in more detailed follow-up with visits at around 6, 12, 18, 24 and 36 months of age [18]. This subsample was recruited specifically to examine patterns and aetiology of childhood obesity in order to aid development of a tailored obesity prevention intervention. 1916 women were eligible to be in this sub-study and 1735 consented and were included. Of these women, 1707 had a singleton birth and 28 had twin births. Analyses presented here are restricted to singleton births only. Infant’s weight and length/height were measured at each follow-up visit, at which time mothers also completed an administered questionnaire which collected information on a range of potentially modifiable risk factors for childhood obesity. Approximately 75% of participants completed each follow-up questionnaire, however not all women completed each questionnaire and data may be missing at one time point but available at subsequent visits. In general women who completed each follow-up visit were more likely to be Pakistani women and older women; there were no differences by maternal education levels or parity. Additional file 1: Table S1 shows the characteristics of those women who did and did not complete the three year follow up visit.

### Outcome measures

Child BMI was calculated from weight and height data collected at the 36 month visit. BMI was converted to age and sex adjusted z-scores relative to the WHO 2006 growth standards [19]. Given the sample size of our study we did not have sufficient power to examine the binary outcome of childhood obesity. We examined associations of modifiable risk factors with two outcomes at age 3: BMI z-scores analysed on a continuous scale and child overweight defined as having a BMI z-score greater than or equal to the 85th centile [20].

### Risk factors

Risk factors were selected if they had been previously shown to be associated with childhood obesity in published literature and it was plausible that they were modifiable [10,11,13,21]. Risk factors that have previously been associated with childhood obesity but were non-modifiable by interventions aimed at the family/child level were not examined. A summary of risk factors considered in the current study, including the time they were collected are shown in Table 1 and described in the following text.

**Table 1:** **Summary of risk factors, method and time of data collection and category level in analysis**

**Variable**	**Method and time of data collection**	**Category level in analysis**
***Pregnancy risk factors***		
Maternal smoking during pregnancy	Self-report at 26–28 weeks gestation	No, Yes
Gestational diabetes	Extracted from maternity notes	No, Yes
Maternal booking BMI	Weight at booking from maternity notes, height measured at 26–28 weeks gestation	Underweight/normal (BMI ≤ 24.9)
		Overweight (BMI 25–29.9)
		Obese (BMI ≥ 30)
***Postnatal risk factors***		
Duration of any breastfeeding	Self-report at 6 and 12 months	Never, 1 day to <1 month, 1 to 4 months, 4+ months
Age at weaning on to solids	Self-report at 6 and 12 months	≥17 weeks, <17 weeks
Infant’s total energy intake per day	Food Frequency Questionnaire (FFQ) at 12 months	Kcals per day: continuous
Infant’s total protein intake per day	FFQ at 12 months	Grams per day: continuous
Caregivers feeding style	Questionnaire at 24 months	Authoritative, Authoritarian, Indulgent, Uninvolved
Parenting style – Parental self-efficacy	Questionnaire at 24 months	High, lower scores (bottom quintile)
Parenting style –Parental warmth	Questionnaire at 24 months	High, lower scores (bottom quintile)
Parenting style – Parental hostility	Questionnaire at 24 months	Low, higher scores (top quintile)
Infant TV viewing time	Questionnaire at 24 months	Hours per day: None, 0-1 hr, 1-2 hrs, 2+ hours
Maternal TV viewing time	Questionnaire at 24 months	Hours per day: 0-2 hrs, 2-4 hrs, 4 + hrs
Total infant sleep duration	Questionnaire at 24 months	Hours per day: <11 hrs, 11-12 hrs, 12-13 hrs, 13 + hrs
Mothers physical activity	Questionnaire at 18 months	Sedentary (no activity), insufficiently active (<150 mins per week), sufficiently active (≥150 mins per week)

Maternal smoking status was coded from self-report at the baseline questionnaire as smoker during pregnancy or non-smoker during pregnancy. Maternal height was measured at baseline and the mother’s booking weight (approximately 12 weeks gestation) was extracted from the hospital maternity IT system. From these, maternal booking BMI was calculated and categorised according to the WHO definitions [22]. Gestational diabetes was defined as either a fasting glucose level ≥ 6.0 mmol/l or a 2-hour postload glucose level of ≥ 7.8 mmol/l, according to WHO criteria [23].

Duration of breastfeeding was ascertained at the 6 and 12 month follow-up visits and from these responses a categorical variable was derived to capture the total duration of breastfeeding for each infant. Information on age at weaning on to solids was collected at the 6 and 12 month visits; an indicator of whether or not this was before 17 weeks (i.e. < 4 months) was created based on current recommendations [24].

Infant dietary data were collected at 12 months using a validated food frequency questionnaire from the Southampton Women’s cohort study [25] which was modified for use in the multi-ethnic population of Bradford. From this, intake of total daily protein (g) and total daily energy (kcal) were calculated.

The caregiver’s feeding styles questionnaire was completed at the 24 month visit [26]. This comprises several questions that measure parental styles of feeding along two dimensions, demandingness and responsiveness, and parents were categorised into one of four feeding styles based on their scores: authoritative (high demandingness/high responsiveness), authoritarian (high demandingness/low responsiveness), indulgent (low demandingness/high responsiveness) and uninvolved (low demandingness/low responsiveness).

Parents self-rated their parenting behaviour when the child was 24 months using questions from another large cohort study in Australia [27], and three domains of parenting practice were derived from the responses; parental self-efficacy, parental warmth and hostile parenting. Most women rated themselves as being self-efficacious, warm and not hostile. Due to this skewed distribution, scores for each domain were summed and divided into quintiles. For parental self-efficacy and parental warmth those in the lowest quintile were classified as having parental self-efficacy scores or parental warmth scores in the lowest quintile. For parental hostility those in the highest quintile were classified as having parental hostility scores in the highest quintile.

Information on infant and maternal TV viewing time was collected at 24 months using questions from the EPIC Norfolk EPIQ-2 questionnaire and coded to calculate an average viewing time per day for both mothers and infants [28].

Infant sleep duration during the day and night was collected at the 24 month visit, from this total sleep duration per 24 hours was calculated.

Mother’s physical activity was measured at 18 months using the methodology of the Active Australia survey [29]. Activity levels were coded into three categories based on self-reported activity time, sedentary (no activity), insufficiently active (<150 minutes of activity per week) and sufficiently active (≥150 minutes of activity per week).

### Other characteristics

Mother’s self-defined ethnicity was collected in the baseline questionnaire and used to define the ethnicity of her offspring using the same ethnic group classification as the 2001 UK Census [30] and categorised into White British, Pakistani and Other. The numbers of participants in the other ethnic groups were too small to analyse separately and were combined.

Highest maternal educational qualification was collected from the baseline questionnaire. Maternal age, infant sex, parity, birthweight, gestational age and mode of delivery were obtained from the hospital maternity system.

### Statistical methods

We examined the distribution of the risk factors for all ethnic groups and then compared the distributions between the White British and Pakistani ethnic groups only, using chi-squared and t-tests as appropriate. We used univariable and multivariable linear regression to examine the association between each risk factor and BMI z-scores. For the binary outcome of child overweight we wished to estimate a risk ratio rather than an odds ratio, which can exaggerate the measure of the strength of association, so we used univariable and multivariable Poisson regression with robust error variances as described by Zou [31] to determine the relative risk between each risk factor and the binary outcome of child overweight. For each outcome and risk factor we considered two models: unadjusted and adjusted for covariables. For pregnancy risk factors this included ethnicity, child sex, maternal education, maternal age and parity. For postnatal risk factors, we additionally adjusted for birthweight, gestational age and mode of delivery. Finally we fitted a fully adjusted model that included all risk factors and covariables. From the fully adjusted multivariable linear regression model we estimated the variance inflation factor (VIF) to assess multicollinearity among the risk factors.

We examined possible ethnic differences in associations with BMI z-scores by repeating the fully adjusted multivariable linear regression analysis for this outcome for the White British and Pakistani groups only (excluding the Other ethnic group) by examining statistical evidence for a difference between the two (i.e. interaction tests between ethnicity and risk factor). From the model including the interaction terms we estimated the mean differences in BMI z-scores separately for the White British and Pakistani groups. We did not conduct these analyses for the binary outcome because of limited statistical power.

BMI data at age 3 were available for 987 children. Of these, complete data on all risk factors and potential confounders were available for 669 participants (68%). We used multiple imputation using chained equations [32] to impute for missing values for risk factors and confounders using 50 imputed data sets. We carried out sensitivity analysis by performing complete case analysis and the results showed similar patterns (results available from author on request).

## Results

Table 2 shows the prevalence of risk factors overall and by ethnic group. On average, compared to Pakistani children, White British children were more likely to have mothers that smoked during pregnancy, have mothers with higher rates of obesity at booking, be breastfed for a shorter duration, be weaned before 17 weeks, have higher daily intake of protein and energy, have mothers with an indulgent caregiver’s feeding style and have mothers that watch more hours of TV per day. Pakistani mothers were more likely to have gestational diabetes, have parental warmth scores in the lowest quintile and be less active than White British mothers, and Pakistani children had greater average TV viewing duration than White British children.

**Table 2:** **Early life risk factors overall and by ethnic group (White British and Pakistani), % or mean (SD)**

**Risk factor**	**All** ^**a**^ **(N = 987)**	**White British (N = 382)**	**Pakistani (N = 474)**	**P** ^**b**^
***Pregnancy risk factors***				
**Smoked during pregnancy (%)**	N = 986	N = 382	N = 474	<0.001
No	85.2	72.3	96.0	
Yes	14.8	27.7	4.0	
**Gestational diabetes (%)**	N = 967	N = 376	N = 461	0.001
No	89.7	92.8	85.9	
Yes	10.3	7.2	14.1	
**Maternal booking BMI category (%)**	N = 949	N = 366	N = 458	0.003
Underweight/normal	51.2	45.1	55.2	
Overweight	30.1	30.9	29	
Obese	18.7	24.0	15.7	
***Postnatal risk factors***				
**Duration of any breastfeeding (%)**	N = 987	N = 382	N = 474	<0.001
Never	27.0	36.1	23.4	
1 day to 1 month	26.3	29.6	26.2	
1 to 4 months	18.5	13.9	21.7	
4+ months	28.2	20.4	28.7	
**Weaned <17 weeks (%)**	N = 924	N = 363	N = 440	<0.001
No	73.8	63.6	81.4	
Yes	26.2	36.4	18.6	
**Daily protein intake, mean (SD)**	N = 866	N = 330	N = 417	0.001
g per day	38.0 (17.2)	40.9 (17.6)	36.5 (17.0)	
**Daily total energy intake, mean (SD)**	N = 866	N = 330	N = 417	0.003
Kcal per day	1105 (433)	1169 (445)	1075 (412)	
**Caregivers feeding style (%)**	N = 904	N = 343	N = 443	<0.001
Authoritative	14.7	15.2	14.4	
Authoritarian	32.4	21.3	39.3	
Indulgent	34.8	48.1	26.0	
Uninvolved	18.0	15.5	20.3	
**Parental self-efficacy (%)**	N = 899	N = 341	N = 440	0.49
High	80.0	80.6	78.6	
Lower scores (bottom quintile)	20.0	19.4	21.4	
**Parental warmth (%)**	N = 897	N = 341	N = 439	0.001
High	73.4	79.8	69.5	
Lower scores (bottom quintile)	26.6	20.2	30.5	
**Hostile parenting (%)**	N = 896	N = 341	N = 439	0.42
Low	76.8	75.7	78.1	
Higher scores (top quintile)	23.2	24.3	21.9	
**Infant TV viewing time (%)**	N = 901	N = 340	N = 443	<0.001
None	7.2	6.5	8.1	
0-1 hour	37.6	44.1	34.1	
1-2 hours	27.0	29.4	23.7	
2-3 hours	28.2	20.0	34.1	
**Maternal TV viewing time (%)**	N = 904	N = 343	N = 443	<0.001
0-2 hours	43.1	33.5	49.4	
2-4 hours	41.9	50.4	36.6	
> 4 hours	14.9	16.0	14.0	
**Infant sleep duration (%)**	N = 898	N = 337	N = 443	0.23
< 11 hours	19.3	17.2	19.0	
11-12 hours	27.6	30.6	25.5	
12-13 hours	30.2	32.3	30.7	
13+ hours	22.9	19.9	24.8	
**Maternal activity level (%)**	N = 902	N = 345	N = 439	<0.001
Sedentary	15.9	9.0	20.7	
Insufficiently active	44.2	33.9	53.1	
Sufficiently active	39.9	57.1	26.2	

^a^This includes 382 White British participants, 474 Pakistani participants and 131 participants of “Other” ethnicities.

^b^Difference between White British and Pakistani (chi squared test or *t*-test as appropriate).

Overall the mean BMI z-score was 0.54 (standard deviation (SD) 1.05) at age 3 and 30% of the children in the study were overweight. BMI z-scores were higher for White British children compared to Pakistani children (mean z-scores 0.74 (SD 0.90) and 0.40 (SD 1.14) respectively) and the percentage of children classified as overweight was also higher (37% for White British and 27% for Pakistani).

Table 3 shows differences in BMI z-scores for each of the risk factors. BMI z-scores were higher in children who had mothers that smoked during pregnancy, were overweight or obese at booking, breastfed between 1 day and 1 month (compared to those who never breastfed), and had an indulgent caregiver’s feeding style, lower parental warmth scores and higher parental hostility scores. BMI z-scores were lower in children of mothers with parental self-efficacy scores in the lowest quintile and mothers who watched more hours of TV.

**Table 3:** **Mean difference in BMI z-score and 95% confidence intervals for each early life risk factors from linear regression models**

**Risk factor**	**Category**	**Model 1** ^**a**^		**Model 2** ^**b**^		**Model 3** ^**c**^	
		**Mean difference**	**95% CI**	**Mean difference**	**95% CI**	**Mean difference**	**95% CI**
***Pregnancy risk factors***							
Smoked during pregnancy	No	0	-	0	-	0	-
	Yes	0.29	(0.11, 0.47)	0.21	(0.01, 0.41)	0.33	(0.13, 0.53)
Gestational diabetes	No	0	-	0	-	0	-
	Yes	−0.13	(−0.35, 0.09)	−0.13	(−0.35, 0.10)	−0.11	(−0.33, 0.11)
Maternal booking BMI	Underweight/normal	0	-	0	-	0	-
	Overweight	0.27	(0.12, 0.42)	0.26	(0.11, 0.42)	0.23	(0.08, 0.38)
	Obese	0.48	(0.31, 0.66)	0.45	(0.27, 0.63)	0.37	(0.19, 0.55)
***Postnatal risk factors***							
Duration of any breastfeeding	Never	0	-	0	-	0	-
	1 day to 1 month	0.22	(0.09, 0.40)	0.23	(0.06, 0.41)	0.22	(0.05, 0.40)
	1 to 4 months	0.08	(−0.12, 0.27)	0.16	(−0.04, 0.36)	0.14	(−0.06, 0.33)
	4+ months	0.04	(−0.14, 0.22)	0.05	(−0.14, 0.23)	0.06	(−0.12, 0.24)
Weaned <17 weeks	No	0	-	0	-	0	-
	Yes	0.13	(−0.02, 0.29)	0.05	(−0.11, 0.20)	−0.05	(−0.21, 0.10)
Daily protein intake	Per 1SD increase	0.08	(0.01, 0.15)	0.05	(−0.02, 0.12)	0.02	(−0.12, 0.15)
Daily total energy	Per 1SD increase	0.08	(0.01, 0.14)	0.05	(−0.02, 0.12)	0.04	(−0.10, 0.17)
Caregivers feeding style	Authoritative	0	-	0	-	0	-
	Authoritarian	−0.11	(−0.32, 0.10)	−0.07	(−0.27, 0.14)	−0.10	(−0.31, 0.11)
	Indulgent	0.24	(0.04, 0.45)	0.19	(−0.01, 0.40)	0.15	(−0.06, 0.36)
	Uninvolved	0.06	(−0.18, 0.29)	0.04	(−0.19, 0.27)	0.05	(−0.18, 0.28)
Parental self-efficacy	High	0	-	0	-	0	-
	Lower scores (bottom quintile)	−0.10	(−0.27, 0.07)	−0.13	(−0.30, 0.04)	−0.18	(−0.35, 0.00)
Parental warmth	High	0	-	0	-	0	-
	Lower scores (bottom quintile)	0.13	(−0.02, 0.29)	0.19	(0.03, 0.34)	0.21	(0.05, 0.36)
Hostile parenting	Low	0	-	0	-	0	-
	Higher scores (top quintile)	0.11	(−0.05, 0.26)	0.09	(−0.06, 0.25)	0.17	(0.01, 0.33)
Infant TV viewing time	None	0	-	0	-	0	-
	0-1 hour	−0.16	(−0.44, 0.11)	−0.18	(−0.45, 0.09)	−0.15	(−0.41, 0.12)
	1-2 hours	−0.06	(−0.35, 0.22)	−0.07	(−0.35, 0.21)	−0.02	(−0.30, 0.25)
	>2 hours	−0.17	(−0.45, 0.11)	−0.11	(−0.38, 0.17)	−0.02	(−0.30, 0.25)
Maternal TV viewing time	0-2 hours	0	-	0	-	0	-
	2-4 hours	0.03	(−0.12, 0.18)	−0.02	(−0.16, 0.13)	−0.08	(−0.23, 0.07)
	>4 hours	−0.26	(−0.46, −0.05)	−0.29	(−0.49, −0.08)	−0.39	(−0.60, −0.18)
Infant sleep duration	<11 hours	0.03	(−0.16, 0.23)	0.10	(−0.10, 0.29)	0.11	(−0.08, 0.30)
	11-12 hours	0.08	(−0.10, 0.26)	0.07	(−0.11, 0.24)	0.04	(−0.14, 0.21)
	12-13 hours	0	-	0	-	0	-
	13+ hours	−0.06	(−0.25, 0.12)	−0.01	(−0.19, 0.18)	−0.02	(−0.20, 0.17)
Maternal activity level	Sedentary	0	-	0	-	0	-
	Insufficiently active	0.06	(−0.13, 0.26)	0.04	(−0.15, 0.23)	0.08	(−0.11, 0.27)
	Sufficiently active	0.20	(0.00, 0.40)	0.07	(−0.13, 0.27)	0.11	(−0.09, 0.31)

^a^Model 1: unadjusted.

^b^Model 2: pregnancy risk factors adjusted for ethnicity, infant sex, maternal age, maternal highest educational qualification, parity. Postnatal risk factors additionally adjusted for birthweight, gestational age at delivery and mode of delivery.

^c^Model 3: adjusted for all risk factors and ethnicity, infant sex, maternal age, maternal highest educational qualification, parity, birthweight, gestational age at delivery and mode of delivery.

The variance inflation factor values in the fully adjusted model were all less than 5 indicating that multicollinearity was not present in these analyses.

Table 4 shows the risk of the child being overweight at age 3 years associated with each of the included risk factors. We found a similar pattern of associations between the risk factors and overweight as we did between the risk factors and mean BMI.

**Table 4:** **Relative risk (RR) and 95% confidence intervals for infant overweight for each early life risk factors from Poisson regression models**

**Risk factor**	**Category**	**Model 1** ^**a**^		**Model 2** ^**b**^		**Model 3** ^**c**^	
		**RR**	**95% CI**	**RR**	**95% CI**	**RR**	**95% CI**
***Pregnancy risk factors***							
Smoked during pregnancy	No	1	-	1	-	1	-
	Yes	1.25	(0.99, 1.60)	1.17	(0.90, 1.52)	1.36	(1.05, 1.76)
Gestational diabetes	No	1	-	1	-	1	-
	Yes	0.98	(0.72, 1.35)	0.96	(0.69, 1.32)	0.98	(0.71, 1.35)
Maternal booking BMI	Underweight normal	1	-	1	-	1	-
	Overweight	1.36	(1.09, 1.71)	1.33	(1.06, 1.67)	1.28	(1.02, 1.61)
	Obese	1.65	(1.30, 2.09)	1.55	(1.21, 1.99)	1.44	(1.11, 1.86)
***Postnatal risk factors***							
Duration of any breastfeeding	Never	1	-	1	-	1	-
	1 day to 1 month	1.40	(1.09, 1.81)	1.42	(1.11, 1.84)	1.38	(1.07, 1.79)
	1 to 4 months	1.10	(0.81, 1.49)	1.21	(0.88, 1.65)	1.16	(0.85, 1.56)
	4+ months	1.05	(0.80, 1.39)	1.07	(0.80, 1.42)	1.08	(0.82, 1.44)
Weaned <17 weeks	No	1	-	1	-	1	-
	Yes	1.04	(0.83, 1.29)	0.96	(0.77, 1.20)	0.86	(0.68, 1.08)
Daily protein intake	Per 1SD increase	1.08	(0.99, 1.18)	1.05	(0.95, 1.15)	1.09	(0.90, 1.30)
Daily total energy intake	Per 1SD increase	1.07	(0.97, 1.18)	1.04	(0.94, 1.15)	0.96	(0.79, 1.16)
Caregivers feeding style	Authoritative	1	-	1	-	1	-
	Authoritarian	0.83	(0.58, 1.19)	0.87	(0.61, 1.24)	0.85	(0.60, 1.22)
	Indulgent	1.42	(1.03, 1.94)	1.34	(0.97, 1.84)	1.29	(0.94, 1.78)
	Uninvolved	1.06	(0.73, 1.53)	1.03	(0.71, 1.49)	1.07	(0.74, 1.55)
Parental self-efficacy	High	1	-	1	-	1	-
	Lower scores (bottom quintile)	0.83	(0.63, 1.09)	0.79	(0.61, 1.03)	0.76	(0.58, 1.00)
Parental warmth	High	1	-	1	-	1	-
	Lower scores (bottom quintile)	1.22	(0.98, 1.51)	1.29	(1.04, 1.60)	1.33	(1.07, 1.65)
Hostile parenting	Low	1	-	1	-	1	-
	Higher scores (top quintile)	1.04	(0.83, 1.31)	1.04	(0.83, 1.31)	1.16	(0.91, 1.48)
Infant TV viewing time	None	1	-	1	-	1	-
	0-1 hour	1.03	(0.68, 1.56)	0.99	(0.65, 1.50)	1.04	(0.69, 1.56)
	1-2 hours	1.08	(0.71, 1.65)	1.08	(0.70, 1.64)	1.19	(0.78, 1.80)
	>2 hours	0.98	(0.64, 1.50)	1.03	(0.67, 1.58)	1.18	(0.78, 1.80)
Maternal TV viewing time	0-2 hours	1	-	1	-	1	-
	2-4 hours	1.00	(0.81, 1.23)	0.96	(0.78, 1.17)	0.88	(0.71, 1.09)
	>4 hours	0.61	(0.42, 0.90)	0.60	(0.41, 0.88)	0.54	(0.37, 0.79)
Infant sleep duration	<11 hours	1.09	(0.82, 1.45)	1.17	(0.87, 1.56)	1.18	(0.88, 1.56)
	11-12 hours	1.05	(0.80, 1.37)	1.04	(0.80, 1.35)	1.00	(0.77, 1.30)
	12-13 hours	1	-	1	-	1	-
	13+ hours	1.05	(0.79, 1.38)	1.12	(0.85, 1.47)	1.13	(0.86, 1.49)
Maternal activity level	Sedentary	1	-	1	-	1	-
	Insufficiently active	0.96	(0.72, 1.30)	0.93	(0.69, 1.25)	0.99	(0.74, 1.33)
	Sufficiently active	1.13	(0.84, 1.51)	0.98	(0.73, 1.32)	1.04	(0.77, 1.40)

^a^Model 1: unadjusted.

^b^Model 2: pregnancy risk factors adjusted for ethnicity, infant sex, maternal age, maternal highest educational qualification, parity. Postnatal risk factors additionally adjusted for birthweight, gestational age at delivery and mode of delivery.

^c^Model 3: adjusted for all risk factors and ethnicity, infant sex, maternal age, maternal highest educational qualification, parity, birthweight, gestational age at delivery and mode of delivery.

Multivariable adjusted associations of each risk factor with BMI z-scores were similar in White British and Pakistani infants (Table 5), and there was no strong statistical evidence that the associations differed by ethnicity with the exception of breastfeeding (all p-values for interaction ≥ 0.1, except p-value for breastfeeding and ethnicity interaction = 0.03). White British mothers who breastfed between 1 and 4 months had infants with lower BMI z-scores, while Pakistani mothers who breastfed for that duration had infants with higher BMI z-scores compared to Pakistani mothers who never breastfed.

**Table 5:** **Mean difference in BMI z-score and 95% confidence intervals for each early life risk factors from regression models with interactions between ethnicity and each risk factor (White British and Pakistani only) fully adjusted model**

**Risk factor**	**Category**	**White British (N = 382)**		**Pakistani (N = 474)**		**Interaction p-value**
		**Mean difference** ^**a**^	**95% CI**	**Mean difference** ^**a**^	**95% CI**	
***Pregnancy risk factors***						
Smoked during pregnancy	No	0	-	0	-	0.12
	Yes	0.37	(0.11, 0.62)	−0.06	(−0.53, 0.42)	
Gestational diabetes	No	0	-	0	-	0.10
	Yes	0.18	(−0.22, 0.58)	−0.23	(−0.51, 0.05)	
Maternal booking BMI	Underweight/normal	0	-	0	-	0.51
	Overweight	0.36	(0.11, 0.60)	0.18	(−0.04, 0.40)	
	Obese	0.34	(0.07, 0.61)	0.35	(0.08, 0.62)	
***Postnatal risk factors***						
Duration of any breastfeeding	Never	0	-	0	-	0.03
	1 day to 1 month	0.09	(−0.17, 0.34)	0.28	(0.02, 0.54)	
	1 to 4 months	−0.24	(−0.57, 0.09)	0.41	(0.13, 0.69)	
	4+ months	0.00	(−0.31, 0.31)	0.15	(−0.11, 0.40)	
Weaned <17 weeks	No	0	-	0	-	0.33
	Yes	−0.09	(−0.32, 0.14)	0.08	(−0.17, 0.33)	
Daily protein intake	Per 1SD increase	0.04	(−0.18, 0.26)	−0.09	(−0.29, 0.12)	0.41
Daily total energy	Per 1SD increase	0.05	(−0.17, 0.27)	0.08	(−0.13, 0.30)	0.83
Caregivers feeding style	Authoritative	0	-	0	-	0.98
	Authoritarian	−0.15	(−0.52, 0.21)	−0.10	(−0.40, 0.20)	
	Indulgent	0.08	(−0.24, 0.39)	0.15	(−0.17, 0.47)	
	Uninvolved	−0.05	(−0.44, 0.35)	−0.04	(−0.38, 0.30)	
Parental self-efficacy	High	0	-	0	-	0.73
	Lower scores (bottom quintile)	−0.25	(−0.53, 0.04)	−0.18	(−0.43, 0.06)	
Parental warmth	High	0	-	0	-	0.73
	Lower scores (bottom quintile)	0.14	(−0.12, 0.41)	0.20	(−0.01, 0.42)	
Hostile parenting	Low	0	-	0	-	0.42
	Higher scores (top quintile)	0.06	(−0.21, 0.32)	0.20	(−0.04, 0.44)	
Infant TV viewing time	None	0	-	0	-	0.56
	0-1 hour	0.13	(−0.32, 0.57)	−0.26	(−0.64, 0.12)	
	1-2 hours	0.23	(−0.23, 0.70)	0.02	(−0.38, 0.42)	
	>2 hours	0.25	(−0.24, 0.74)	−0.05	(−0.43, 0.33)	
Maternal TV viewing time	0-2 hour	0	-	0	-	0.10
	2-4 hour	0.09	(−0.16, 0.33)	−0.22	(−0.44, −0.01)	
	>4 hour	−0.43	(−0.77, −0.09)	−0.40	(−0.70, −0.10)	
Infant sleep duration	<11 hours	0.08	(−0.23, 0.39)	0.12	(−0.16, 0.39)	0.97
	11-12 Hours	0.00	(−0.27, 0.28)	0.09	(−0.16, 0.35)	
	12-13 Hours	0	-	0	-	
	13+ hours	−0.05	(−0.35, 0.25)	0.03	(−0.23, 0.28)	
Maternal activity level	Sedentary	0	-	0	-	0.75
	Insufficiently active	0.18	(−0.22, 0.58)	0.02	(−0.22, 0.26)	
	Sufficiently active	0.16	(−0.23, 0.55)	0.09	(−0.19, 0.38)	

^a^Adjusted for all risk factors and infant sex, maternal age, maternal highest educational qualification, parity, birthweight, gestational age at delivery and mode of delivery.

## Discussion

In this bi-ethnic study we found that the prevalence of early life risk factors for childhood obesity differed between mothers of White British and Pakistani ethnicity. However, the associations between these risk factors and BMI at age 3 were similar in the two ethnic groups. Undertaking analyses on both ethnic groups combined, both mean BMI and the relative risk of being overweight were greater at age 3 in children whose mothers had higher BMI at booking clinic, smoked during pregnancy, had more indulgent feeding style and a more hostile or less warm parenting style. Mean BMI and the relative risk of being overweight were lower in children with mothers who reported lower parental self-efficacy and mothers who watched more hours of TV.

Consistent with other studies, we found that children of mothers who smoked during pregnancy were more likely to have higher BMI z-scores and greater risk of overweight [10-12], as were children of overweight and obese mothers [11,13]. Whether this association is causal or not is unclear. Studies comparing maternal smoking with offspring BMI or overweight, to the same association of paternal smoking with this outcome, have found the two to be similar, suggesting that the maternal association may reflect confounding by shared familial characteristics [33-35]. This is also supported by a recent within sibling study [36].

We found some aspects of feeding and parenting style were associated with the child’s BMI. Parents with an ‘indulgent’ feeding style had children with higher BMI z-scores and more likely to be overweight. An indulgent feeding style is a permissive feeding style that uses less controlling feeding practices. These findings are consistent with other studies which have also found that children of indulgent parents had higher BMI z-scores [16,26]. We also found that children of parents with lower parental warmth and higher hostile parenting styles were more likely to be overweight. In contrast infants of parents with lower self-efficacy scores had lower BMI z-scores and reduced risk of being overweight. Different measures and constructs of parenting styles have been studied in the literature, however evidence linking the specific domains analysed in our study to childhood BMI is scarce. Some studies have looked at the associations with dietary and activity behaviours [15,37] and evidence suggests that children raised by authoritative parents had lower BMI levels compared to children who were raised with other styles [15]. The evidence evaluating the relationship between maternal self-efficacy and child weight outcomes is scarce, although, evidence from other studies does suggest that greater maternal self-efficacy has a protective effect against obesity related behaviours [37-39]. Many of these studies are cross-sectional, conducted on small samples or on children who are overweight or obese; therefore further replication of our findings in other longitudinal studies are needed.

The association of breastfeeding with childhood BMI has been examined in a number of observational studies. Systematic reviews suggest a protective effect of breast feeding, but with heterogeneity between studies [10,11,40]. We did not find a protective association of breastfeeding in our study, and indeed found that mean BMI and risk of overweight were greater amongst infants who were breastfed in the first year of life compared to those who were never breastfed, and some evidence that these associations differed by ethnic group. Randomised controlled trial evidence and cross-cohort comparisons suggest that the observational associations of breast feeding with BMI are not causal [41,42], and it is possible that our findings represent random variation around a true null association that has to date has not been apparent in systematic reviews as these have largely been conducted using observational study designs limiting causal inferences and perhaps reflecting publication bias.

We found that children of mothers who watched more hours of TV had lower BMI z-scores and a reduced risk of being overweight, however there was no strong evidence of an association between child TV viewing time and BMI. Previous research has shown a positive association between child TV viewing time and childhood overweight and obesity [13]; however little research has been conducted in this young age group, or investigating the association between maternal viewing and child weight outcomes. Our findings may be due to unmeasured confounding, reverse causality or measurement error. There are difficulties in accurately measuring screen time in children and accurate measurement to predict the total number of minutes of TV time per day may require a more comprehensive, prospective questionnaire or diary [43], which was not feasible in this cohort. TV viewing is only one aspect of total sedentary time which includes behaviours such as computer use, time spent sitting at work, during transport and in leisure time; relationships between sedentary behaviour and health are unlikely to be explained using single markers of inactivity [44]. Recent evidence from a meta-analysis of several studies has shown no independent association between sedentary behaviour and child BMI [45].

In this study we did not find strong evidence of an association between gestational diabetes, age at weaning, infant energy intake, infant protein intake, child sleep duration and mean BMI or risk of overweight at age 3 despite evidence of associations between these risk factors and BMI in other studies [10,11,14]. However many of these studies were conducted in older age groups and in non-UK populations with different ethnic compositions to the Born in Bradford study population.

### Strengths and limitations

The main strength of this paper is that we have considered several key modifiable risk factors that have been collected longitudinally during pregnancy and early childhood in a bi-ethnic cohort. To our knowledge this is the first time these risk factors have been studied in early life in children of Pakistani origin. We were able to assess the associations between the risk factors and BMI z-scores separately in the White British and Pakistani groups, in addition to adjusting for several important maternal and child characteristics in our models. We used multiple imputation techniques to improve the integrity of our results as complete data were only available for 68% of our sample; results for the imputed analyses and complete case analyses showed similar patterns.

One of the limitations of our analyses is multiple testing, we considered many risk factors and may have found some associations to be statistically significant through chance alone, however in reporting our results we have focused on the magnitude and direction of the effect sizes and not only on the p-values.

We were unable to assess whether associations of the binary outcome of overweight differed by ethnic group due to limited statistical power. However, for the main analyses it can be seen that associations with BMI z-score as a continuous variable and with it split into a binary variable of overweight or not, are consistent with each other and therefore the finding that, in general, associations are consistent in the two groups for BMI z-score suggests they are also likely to be so for overweight. We also had inadequate statistical power to examine associations with obesity separate from overweight. Though, again given the associations with BMI z-scores, we assume that for risk factors found to be associated with increased BMI z-score and overweight, they would also be associated with increased risk of obesity. Our analyses are observational and we cannot assume causality for any of the associations we have found.

Several of the risk factors were collected at multiple time periods during early childhood, however it was not clear which period is the most influential, and this may differ for each risk factor. The correlations between the risk factors collected over time were low and the distributions of these risk factors changed between 6 months and 2 years. We used the risk factor collected closest in time to the outcome as we were unable to derive a meaningful average over early childhood. Our results may be influenced by reverse causality, although we considered risk factors collected up to age 2 and outcomes at age 3 so this is unlikely.

Further follow-up of this cohort to examine the relationship between these risk factors and longer term health outcomes is important.

## Conclusion

In conclusion, whist the prevalence of risk factors associated with increased childhood BMI differed between White British and Pakistani groups the magnitude of their associations with BMI were similar in the two ethnic groups. This work adds to the literature on the association of pregnancy and early life exposures and later childhood BMI and may be useful to identify suitable targets for obesity prevention interventions in pregnancy and early childhood.

## Additional file

Additional file 1: Table S1. Comparison of those that completed and those that did not complete the three year follow-up visit.

## Abbreviations

BMI: Body mass index
VIF: Variance inflation factor
RR: Relative risk
