# Supplementary material for: The relationship between early life modifiable risk factors for childhood obesity, ethnicity and body mass index at age 3 years: findings from the Born in Bradford birth cohort study
Source: BMC Obes. 2015 Feb 24;2:9. doi: 10.1186/s40608-015-0037-5 (PMC4510905; doi:10.1186/s40608-015-0037-5)
Supplement: Additional file 1: Table S1. — Comparison of those that completed and those that did not complete the three year follow-up visit. [file 40608_2015_37_MOESM1_ESM.doc]

**Additional file 1**

Table S1: Comparison of those that completed and those that did not complete the three year follow-up visit

| **Variable** | **Completers**  **N=1200** | | **Non-completers**  **N=507** | |
| --- | --- | --- | --- | --- |
|  | **n** | **%** | **n** | **%** |
| **Ethnic group** |  |  |  |  |
| White British | 450 | 37.5 | 201 | 39.6 |
| Pakistani | 587 | 48.9 | 221 | 43.6 |
| Other | 161 | 13.4 | 81 | 16.0 |
| Missing | 2 | 0.2 | 4 | 0.8 |
| **Mother’s education** |  |  |  |  |
| <=5 GCSE equivalent* | 644 | 53.7 | 287 | 56.6 |
| >=A-level equivalent** | 457 | 38.1 | 180 | 35.5 |
| Other*** | 75 | 6.3 | 28 | 5.5 |
| Missing | 24 | 2.0 | 12 | 2.4 |
| **Maternal age** |  |  |  |  |
| Mean (SD) | 27.5 | 5.7 | 26.3 | 5.6 |
| **Parity** |  |  |  |  |
| Primiparous | 452 | 37.7 | 201 | 39.6 |
| Multiparous | 719 | 59.9 | 293 | 57.8 |
| Missing | 29 | 2.4 | 13 | 2.6 |

GCSE = General Certificate of Secondary Education. Qualifications usually taken at 16 years (legal minimum school leaving age in the UK)

A-level = Advanced level. Qualifications usually taken at age 16-18 before attending university.

Other = Other qualifications (e.g. City and Guilds, RSA/OCR, BTEC) and don’t know responses
